# Lignin Biosynthesis and Its Diversified Roles in Disease Resistance

**DOI:** 10.3390/genes15030295

**Published:** 2024-02-25

**Authors:** Qing-Hu Ma

**Affiliations:** State Key Laboratory of Plant Diversity and Specialty Crops, Institute of Botany, Chinese Academy of Sciences, Beijing 100093, China; mqh@ibcas.ac.cn

**Keywords:** lignin, defense response, biosynthesis, disease resistant crop, molecular breeding

## Abstract

Lignin is complex, three-dimensional biopolymer existing in plant cell wall. Lignin biosynthesis is increasingly highlighted because it is closely related to the wide applications in agriculture and industry productions, including in pulping process, forage digestibility, bio-fuel, and carbon sequestration. The functions of lignin *in planta* have also attracted more attentions recently, particularly in plant defense response against different pathogens. In this brief review, the progress in lignin biosynthesis is discussed, and the lignin’s roles in disease resistance are thoroughly elucidated. This issue will help in developing broad-spectrum resistant crops in agriculture.

## 1. Introduction

Lignin is a main structural component of cell walls in vascular plants (pteridophytes, gymnosperms, and angiosperms) during the process of thickening the secondary wall. Lignin is principally deposited in the secondary wall of certain plant tissues, including xylem, sclerenchyma, phloem fiber, and periderm, which are involved in hydrophobic protection and the mechanical support of plant tissues. Some parenchyma cells may also have the lignin deposition in the primary wall, but the degree of lignifications is low. Lignin is linked to cellulose and hemicellulose in the cell wall to form an extracellular matrix. This structure increases the mechanical intensity and supportable ability of plant tissues. It contributes to rigidity and strength of the plant stem, which is related to the lodging-resistant and seed-coat-protecting phenotypes in crop plants [1,2,3]. Because the natural property of hydrophobicity, lignin imparts water impermeable plant cells. This function is very important, not only for xylem and phloem to transport water and mineral components, but also for the successful colonization of land by plants. In fact, the terrestrial vascular plants are proposed to evolve on the earth by the concomitant evolution of lignin biosynthesis that originated about 450 million years ago in the Silurian Period [4]. Therefore, lignin is a substance unique to vascular plants such as pteridophytes, gymnosperms, and angiosperms. The unicellular and non-vascular plants, such as algae and bryophytes, do not contain the cells filled with lignin, but they do have some lignin biosynthesis-related genes [5]. In addition, lignin accumulation in the cell wall forms a physically structural barrier to effectively protect the plant from pathogens, and the lignin synthesis is induced in response to various kinds of abiotic and biotic stresses [6,7]. We will discuss this topic in later sections.

Second only to cellulose, lignin is one of the most plentiful biopolymers on earth, which accounts for about 1.4 × 10^12^ kg of carbon fixed into terrestrial plants annually [8]. Lignin content varies among different classes of plants. In trees, lignin content represents 27–32% of dry weight, while it accounts only 14–25% of dry weight in herbaceous plants. As a bio-undegradable biopolymer in plant, lignin is also intimately related to industry and agriculture. Lignin content and composition are limiting factors associated with both the quality of paper production and the digestibility of forage crop. In the pulping process, lignin must be removed by costly and environmentally hazardous protocols, spending large amounts of energy and chemicals which may lead to serious environmental pollution. It would be beneficial to treat plant tissues with either less lignin or lignin with an altered chemical reactivity [9]. Lignin content in forage crops is negatively corrected with forage digestibility for ruminant animals, while lignin composition also affects the forage digestibility. Increasing the digestibility of forage crop is important in husbandry [10]. Lignin is also an important determinant in bio-fuel production. The value of lignin depends on its final purpose of utilization. Due to its high heat value, lignin is desirable for conversion by gasification or pyrolysis to produce bio-oil plus useful gas such as H_2_ and CH_4_ [11]. Conversely, ethanol production with enzymatic saccharification in lignocellulose is restricted by lignin-derived compounds [12,13]. Lignin-derived monomers are valuable precursors to produce aromatic chemicals in the biorefinery [14,15]. In the terrestrial environments, lignin is a main component of organic substance that served as the important carbon sink in carbon sequestration [16].

In this review, we will not discuss every aspect in the lignin research field. Instead, we focus on lignin’s actions on disease resistance, which have made comprehensive progress recently.

## 2. Unique Features of Lignin Biosynthesis

**Figure 1:**
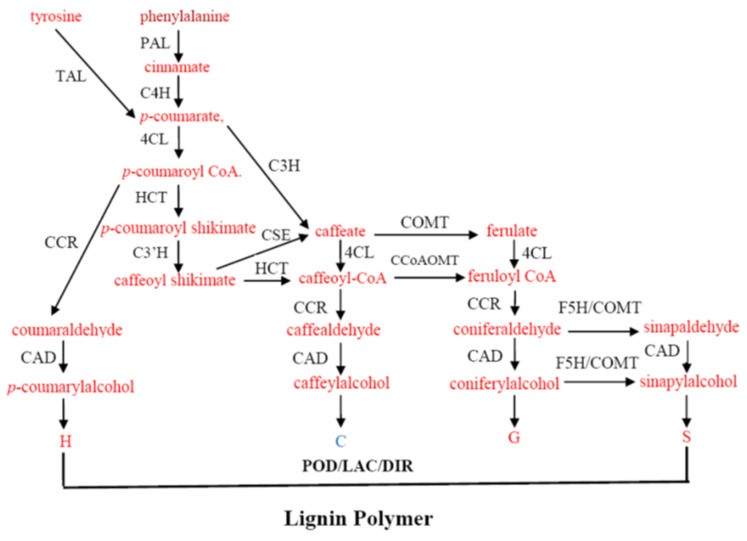
The general biosynthesis pathway of lignin. PAL: phenylalanine ammonia-lyase; TAL: tyrosine ammonia-lyase; C4H: cinnamate 4-hydroxylase; C3H: *p*-coumarate 3-hydroxylase; C3′H: coumaroyl shikimate3′-hydroxylase; 4CL: 4-coumarate: CoA ligase; HCT: hydroxycinnamoyl-CoA shikimate/Quinate hydroxycinnamoyl transferase; F5H: ferulate 5-hydroxylase; CSE: caffeoyl shikimate esterase; CCR: cinnamoyl-CoA reductase; CCoAOMT, caffeoyl-CoA *O*-methyltransferase; COMT: caffeic acid *O*-methyltransferase; CAD: cinnamyl alcohol dehydrogenase; LAC: laccase; POD: peroxidase; DIR: dirigent; H: hydroxyphenyl lignin; C: catechyl lignin; G: guaiacyl lignin; S: syringyl lignin.

Lignin biosynthesis is a complex biochemical pathway which is initiated in deamination of L-phenylalanine or tyrosine into cinnamic acid, then a series of hydroxylation and methylation reactions convert cinnamic acid into a variety of hydroxycinnamic acids. These hydroxycinnamic acids act as precursors not only for lignin, but also for flavonoids. Activation of hydroxycinnamic acids to their corresponding co-enzyme A (CoA) thioesters, followed by successive reductions, produce monolignols which are thought to build into the lignin polymer by polymerization (Figure 1). This conventional pathway of lignin biosynthesis, introduced in the 1980s, has much modification after extensive research in biochemistry, molecular biology, and genetics [17,18,19]. A complete enumeration of each step in lignin biosynthesis is considerably beyond the scope of this review. Here, we just list some important achievements in recent years.

Generally, lignin is derived from three major hydroxycinnamyl alcohols (monolignols), namely *p*-coumaryl alcohol, coniferyl alcohol, and sinapyl alcohol, which convert into *p*-hydroxyphenyl (H), guaiacyl (G), and syringyl (S) lignin, respectively. In addition, caffeyl alcohol (forming catechyl lignin, C) has been found naturally in some species [20]. 5-hydroxyconiferyl alcohol forms a 5-hydroxyguaiacyl (5HG) unit that arises from the transgenic plants by down-regulated caffeic acid *O*-methyltransferase (COMT) and also occurs naturally in the cactus seed coat [21]. Ubiquitous existence of these non-canonical monolignols still requires more investigations [19]. Tricin, a flavone compound, has shown to be present in all grass lignin, where it is suggested to serve as an initiator for polymerization [22,23].

New enzymes have been identified recently in the lignin pathway. Caffeoyl shikimate esterase (CSE) has been demonstrated to convert caffeoyl shikimate (and to a lesser extent coumaroyl shikimate) into the free acid [24]. This fills the gap for a series of reactions termed ‘shikimate shunt’ or ‘esters pathway’ that involve hydroxycinnamoyl-CoA:shikimate hydroxycinnamoyl transferase (HCT) and coumaroyl shikimate3′-hydroxylase (C3′H). This strengthens the cross-talking between the phenylpropanoid pathway with shikimate and the aromatic amino acid biosynthesis. The real biological significance remains open to be investigated. The functions of some old enzymes have also been revised. It was known that L-tyrosine ammonia-lyase (TAL) activity was present in monocot plants. A recent report has shown that purple false brome (*Brachypodium distachyon*) possesses 8 phenylalanine ammonia-lyase (PAL) genes. One of them encodes a bi-functional PAL/TAL (PTAL). This PTAL is preferentially involved in S lignin synthesis. Isotopic labeling experiments have shown that approximately 50% of the lignin is synthesized from L-tyrosine rather from L-phenylalanine [25]. Ferulate 5-hydroxylase (F5H) is a cytochrome-P450-dependent monooxygenase that was originally thought to catalyze the hydroxylation at the C_5_ position of ferulic acid to form 5-hydroxyferulic acid, which was the precursor to S lignin. However, a serial of work from the transgenic plants, feeding tests, and *in vitro* biochemistry have demonstrated that F5H actually used coniferaldehyde and coniferyl alcohol to form 5-hydro-coniferaldehyde and 5-hydro-coniferyl alcohol then syringyl alcohol. Over-expressing F5H in transgenic tobacco and poplar gave rise to lignin that was composed of almost S units [26,27,28]. Therefore, F5H is now also called coniferaldehyde 5-hydroxylase (CAld-5H) [29]. Furthermore, F5H in lycophytes can catalyze S lignin synthesis directly from *p*-coumaraldehyde and *p*-coumaryl alcohol, which is different to angiosperms [30,31].

The monolignols are polymerized into high molecular weight lignin that catalyzed with laccases (using O_2_) and peroxidases (using H_2_O_2_). Both laccases and peroxidase have many isoforms that encoded by large gene families, for instance, *Arabidopsis* contains 17 laccase genes and 73 class III peroxidase genes [32,33]. The exact roles for each member of laccases and peroxidase in monolignol polymerization are still not quite clear. Some reports showed that laccase might be indispensable in initiating lignification of vascular tissues [34,35,36]. It was proposed that laccase might be important in the initiation stage, while peroxidase plays the roles in the bulk polymerization of lignin [37,38]. However, recent findings showed that peroxidase was also essential for lignin polymerization in Casparian strip in root tissues [39].

Dirigent (DIR) is a new class of proteins which were first isolated from weeping forsythia (*Forsythia suspense*) [40]. It has been shown that DIR protein, in the presence of an oxidase or one electron oxidant, can stereo-selectively couple two coniferyl alcohol molecules into a (+)-pinoresinol. This dimer, known as lignan, was presumed to couple more monolignols and then formed lignin polymer. However, direct evidence to support DIR’s roles in lignin biosynthesis is still rare, until recently. A DIR protein, namely ESB1 (enhanced suberin 1), has been shown to play an essential role for the correct formation of lignin in Casparian strip [41]. Moreover, the sub-class of DIR, namely DIR-E, including ESB1, has been demonstrated to be essential for both the localized lignin polymerization required for Casparian strip biogenesis and for the attachment of this strip to the plasma membrane to seal apoplast [42]. An enduring mystery remains that many other members of DIR have not been involved in lignin synthesis, instead promoting lignan synthesis and increase stress response in plants [43,44]. Some DIRs can fuse with other proteins to form chimeric proteins. An interesting example is DIR fused with jacalin to form monocot chimeric jacalin, a novel subfamily of lectins [45,46]. An enigma remains whether DIR is involved in lignin polymerization outside of the Casparian strip. Therefore, two hypotheses of random coupling and strict regulation to address how connecting monolignol radical to produce a functional lignin molecule are still in debate [47,48].

There are some unique features of lignin which are distinct from other macromolecules such as protein and cellulose.

(1). Heterogeneity. Lignin is synthesized from three main monolignols, namely *p*-coumaryl alcohol, coniferyl alcohol, and sinapyl alcohol. This forms hydroxyphenyl (H), guaiacyl (G) and syringyl (S) units in lignin polymers, respectively. In addition, there are various kinds of intermonomeric linkages with up to 16 types in theory in the lignin molecule [49]. The most frequent inter-unit linkage is the C-O-C_4_ (β-aryl ether) linkage, and it is also the one most easily degraded chemically. The other common linkages are the C-C aryl linkage, including C-C_5_, C-C_1_, C-C, and C_5_-C_5_, which are all more resistant to chemical degradation. Lignin has diverse compositions in various plant taxons. Gymnosperms contain mainly G lignin, dicot plants contain G and S lignin, while monocot plants have the lignin composed of G-, S-, and, H-monomers in various ratios [50]. For example, pine trees contain G lignin, and tobacco has an almost similar amount of G and S lignin, while wheat lignin constitutes approximately 50% S plus 40% G and 10% H units. The composition of lignin will also change upon different tissues and developmental stages of plants. This makes the elucidation of the lignin’s functions more sophisticated.

(2). Having properties both for primary and secondary metabolism. On the one hand, lignin is an indispensable and important component of plant cell walls, while on the other hand, lignin belongs to the phenylpropanoid pathway including flavonoids and lignans, which is the typical secondary metabolism.

(3). Contribution to plant development and defense response. Lignin has a crucial role in plant growth and development; it exhibits ubiquitous synthesis in the middle and later stages of plant development. Also, lignin’s synthesis is inducible upon pathogen invasion in the specific tissues which confer resistance to associated pathogens. How to coordinate lignin’s roles in these different aspects remains an enduring mystery. This issue will be discussed more comprehensively in the following section.

## 3. Multifarious Functions of Lignin in Plant Defense Responses

There is a myriad of documents reporting lignin’s involvement in plant disease resistance. To give a clear picture, we strive to sort out these vast data according to their different mechanisms.

### 3.1. Lignin as the Critical Barrier Contributing to Basic Disease Resistance

Lignin is an intricate polymer that serves the physical barrier in the defense response to pathogen infection, as lignin is un-degradable to most microorganisms [51,52]. When pathogens invade a cell, they induce lignin deposition in the cell wall which provides a physical barrier to resist pathogen infection by limiting the entry of pathogen toxins and cell wall-degrading enzymes into plants and preventing the nutrient transmission from the host to the pathogen [53,54]. It is important to know how lignin accumulation will affect disease resistance in plants. The majority of data shows that the high lignin levels will increase disease resistance, but contrary results were also reported that low lignin content in plants exhibited less disease severity [55,56,57]. As a highly labile heteropolymer, lignin composition was also proposed to affect the disease severity in plant. Here, the data showed that S, G, or H lignin might affect disease severity. More G and H units were accumulated when soft rot pathogens infected in Chinese cabbage [58]. The S unit concentration was increased in false flax (*Camelina sativa*) and wheat upon fungal penetration [53,59]. The contradictory results mainly derive from the different plants in study, which are complicated by the many unrestrained genetic and developmental factors possibly impacting defense responses.

In an array of transgenic tobacco plants with modified lignin content or composition by altering single gene, we examined the relationship between lignin content, composition, and disease resistance. The results showed that lower total lignin content aggravated the disease severity, while increased S lignin alleviated the disease symptoms. Neither G nor H lignins exhibited any influences on disease resistance. These data suggest that both total lignin content and S lignin are the main factors that are involved in basic defense response [60]. This sheds light on the complexity of lignin’s connection with plant defense response.

### 3.2. Lignin Related Chemicals Inducing Immune Reaction

Lignin and some related compounds can play as a signal to activate plant-specific immune response. It has reported that silencing Gh4CL30 will promote caffeic acid and ferulic acid accumulation, which inhibit the growth of fungal hyphal and increase resistance to Verticillium wilt in cotton [61]. Many molecules associated with the lignin pathway can serve as phytoalexins which restrict pathogens [62]. Coumarins (including umbelliferone, esculetin, and scopoletin) are synthesized through *p*-coumaryl-CoA and feruloyl-CoA. They have been proposed to be regulators in plant microbiomes [63]. Stilbenes are phenolic phytoalexins. Its skeleton (stilbene skeleton) synthesis is catalyzed by stilbene synthase (STS) through the conversion of *p*-coumaryl-CoA. The defensive roles of stilbene against pathogens have also been documented [64]. Recently, a large-scale and in-depth investigation of the phyllosphere microbiome in rice has revealed that 4-hydroxycinnamic acid (4-HCA), a precursor compound in lignin synthesis, is the main driver for enrichment of beneficial *Pseudomonas*, and inhibition of harmful bacteria *Xanthomonas*. OsPAL02 is responsible for 4-HCA synthesis, and therefore maintains healthy phyllosphere homeostasis in rice. It is proposed that regulating microbiome-shaping genes become a new strategy as ‘M gene breeding’ in plant disease resistance breeding alone with the current strategy known as ‘*R* gene breeding strategy’ [65].

Lignans are phenylpropanoid dimmers synthesized via the monolignol pathway, with coniferyl alcohol as the direct precursor [66]. Dirigent proteins have been shown to act in initiating lignan synthesis [44]. Both dirigent and lignan are proposed to have vital roles in defense responses [67,68,69]. Particularly, some dirigent proteins boost disease resistance by directly promoting lignan accumulation [70].

Besides these lignin compounds’ ability to act directly on pathogens, cell wall damage will affect cell wall integrity (CWI) and then release damage-associated molecular patterns (DAMPs) which trigger immunity reactions [71]. Lignin is proposed to play the critical part during this process [72]. The reactive oxygen species (ROS) and stress-related hormones, such as jasmonate (JA) and salicylic acid (SA), are involved in lignin’s action to disease resistance [73]. A dirigent protein DIR7 has been identified which play the important role in response to plant CWI impairment [74]. Blue copper binding (BCB) protein is involved in electron transfer during oxidative stress response. A BCB, namely GhUMC1, has been demonstrated to increase cotton resistance through H_2_O_2_, JA signaling, and lignin metabolism [75]. It remains uncertain that lignin-specific molecules trigger a burst of ROS or ROS-strength lignin deposition. It has been proposed that polymerizing monolignol into lignin required hydrogen peroxide which is a detoxification process for ROS. Some enzymes in the monolignol pathway are linked to this mechanism, including *p*-coumarate 3-hydroxylase (C3H), CSE, and cinnamoyl-CoA reductase (CCR) [19]. Alternatively, ROS is a signal that directly plays a role in stress responses [76].

### 3.3. Lignin Related Genes Serving Target in Defense Response

In plants, resistance genes (*R*) play a vital part in disease resistance. Most *R* genes encode the NLR class of proteins [77]. Upon pathogen recognition of *R* genes, it triggers a defense response that includes hypersensitive response (HR). HR leads a rapid cell death in infection site. It has been reported that maize has two NLRs, Rp1-D, and Rp1-dp2. Combination of Rp1-D and Rp1-dp2 will lead to activated HR without pathogen infection. Two key enzymes in lignin biosynthesis, HCT and caffeoyl CoA *O*-methyltransferase (CCoAOMT), have been demonstrated to suppress this HR by interacting with the Rp1-D21 complex. The enzymatic activities of HCT and CCoAOMT are not necessary to suppress HR. It is proposed that HCT, CCoAOMT, and Rp1 proteins form a complex. Pathogen effectors may target on the lignin pathway as its importance to plant defense, in turn, NLR proteins will monitor special components during this process [78,79]. This model is reminiscent to resistosome, which has been elucidated recently [80].

Pathogenesis is also involved in lignin by targeting its synthetic enzymes. An F-box protein (ZmFBL41) has been identified that confers resistance to banded leaf and sheath blight (BLSB) in maize. ZmFBL41 interacts with cinnamyl alcohol dehydrogenase (CAD), the final enzyme in the monolignol pathway, leading to the ubiquitination and degradation of CAD. Two amino acid substitutions in the natural allele of resistant maize lines prevent this interaction. It is proposed that the pathogen (*Rhizoctonia solani*) may deliver effectors to directly or indirectly interact with ZmFBL41 or ZmFBL41-ZmSKP1-ZmCAD complex and increase susceptibility of the host [81]. The protein containing tetratrico-peptide repeats (TPRs) is the largest functional family that maintains protein organization and homeostasis through a complicated chaperone network [82]. A mutant, namely *bsr-k1* (broad-spectrum resistance Kitaake-1), has been identified in rice. *Bsr-k1* confers broad-spectrum resistance against the fungal pathogen (*Magnaporthe oryzae*) and bacterial pathogen (*Xanthomonas oryzae*). *Bsr-k1* encodes a tetratricopeptide repeats (TPRs)-containing protein, which binds to PAL mRNAs (OsPAL1-7) and promotes their turnover. Loss of Bsr-k1 function results in lignin accumulation and increases resistance to rice blast and bacterial blight [83].

### 3.4. The Regulating Network Linking Lignin with Immune Reaction

The transcriptional regulation on plant metabolism and development is important, which also participates in immune reaction through lignin metabolism. MYB proteins are one of the largest transcription factor families which play an important part in plant growth and development. Some members of MYB are master regulators in the lignin pathway, usually form MBW ternary complex that consists of MYB, basic helix-loop-helix, and WD40 [84,85]. A R2R3 MYB transcription factor, namely GhODO1, was isolated from cotton. GhODO1 interacts with the promoters of lignin genes Gh4CL1 and GhCAD3, activates their expression, and increases lignin accumulation and resistance to Verticillium wilt (*Verticillium dahlia*). JA-mediated defense signaling is also proposed to be involved in this process [86]. AtMYB15 has been reported to regulate defense-induced lignification and contribute to resistance to *Pseudomonas syringae* (Pst DC3000). Furthermore, effector-triggered immunity (ETI) responses to Pst DC3000 challenge are required for AtMYB15-mediated lignification. This suggests that MYB15 plays a central part in pathogen-induced lignification [87,88]. BnMYB43 from oilseed rape has been shown to regulate vascular lignification, plant morphology and potential yield, but negatively affect resistance to *Sclerotinia sclerotiorum*, therefore being a growth-defense trade-off participant [89]. Recently, an ethylene response factor (ERF) MdEFR114 has been shown to interact with R2R3-MYB and WRKY transcription factors. This complex will directly bind to a peroxidase promoter and increase lignin accumulation and resistance to replant disease in apple tree [90].

Small GTP-binding proteins exist ubiquitously in eukaryotes, which regulate different cell functions such as organogenesis, polar growth, cell division, and defense response [91,92]. ROP is a subfamily of small GTP-binding proteins that exclusively occur in plants. There are 11 ROPs in *Arabidopsis*, 7 in rice, and 6 in wheat [93]. OsRac1, one member of ROP in rice, has been reported to affect on CCR, the first enzyme special to lignin monolignol pathway, and then increase defense responses [94]. Furthermore, we have shown that wheat TaRac1 interacted with TaCCR, up-regulated CCR, and CAD gene expression, enhanced total lignin accumulation and S lignin proportion. This will increase broad-spectrum disease resistance. Collective data suggest that only Group II ROPs have the important roles during defense response in monocot crop [95]. AtRop9, the dicot plant homologue of Group II, is the regulator on ABA and auxin signaling in embryo and lateral root development instead of pathogen response [96]. It is proposed that some ROPs will form a protein network called the defensome, in which ROP serve as a molecular switch to interact with many different effector proteins [97,98]. This bears a resemblance to resistosome complex that was reported recently [80].

### 3.5. The Metabolic Flux towards Lignin Affecting Defense Response

The metabolic reprogramming is a common phenomenon in regulating metabolism of plant. Its relation with plant innate immunity and lignin pathway remain largely unknown. A novel glycosyltransferase UGT73C7 was identified from *Arabidopsis*. It has shown that UGT73C7 could glycosylate *p*-coumaric acid and ferulic acid, the upstream compounds in the lignin pathway. This will up-regulate SNC1 expression, a Toll/interleukin 1 receptor-type *NLR* gene, and then activate immunity in the plant. UGT73C7 is an important regulator to redirect lignin metabolism upon pathogen challenge [99]. Recently, we have demonstrated that wheat DFRL exerted disease resistance through shifting NADP pool and lignin synthesis [100]. *Hm1* is a first-cloned *R* gene from maize, which encodes an enzyme that detoxifies the *Helminthosporium carbonum* (HC) toxin from the special pathogen *Cochliobolus carbonum* [101]. However, the homologous *Hm* genes have also been found from other monocot crops, including rice, barley, and wheat, although they are not the host of *C. carbonum*. *Hm* homologs are similar with dihydroflavonol-4-reductase (DFR) in sequence, an important rate-limiting enzyme in flavonoid pathway; therefore they are named as dihydroflavonol-4-reductase like (DFRL). Our studies have shown that wheat TaDFRL has the broad substrate preference, including dihydroflavonol (such as taxifolin), flavonol, and flavones (such as quercetin and apigenin), and use both NAD and NADP as co-enzyme, which is different with DFR. Up-regulated *TaDFRL* alters NAD(H) and NADP(H) pools towards high NADPH levels. Subsequently, the expressions of CAD and CCR genes are increased, which required NADPH as reducing equivalent. This leads to the enhancement of lignin accumulation and resistance to broad-spectrum diseases [100]. This provides a novel mechanism about increasing host defense responses by elevating metabolic flux towards lignin biosynthesis.

## 4. Conclusions and Perspectives

The increasing reports on lignin’s role in disease resistance have been shown in the recent scientific literature. As we discuss in this review, many questions remain to be solved on this issue. The heterogeneity in lignin synthesis and the different stress-induced lignin to developmental lignin make our understanding on the relationships between lignin and defense more sophisticated, which lead to many conflicting reports. Therefore, it is reasonable to excavate and distinguish the different mechanisms behind these actions in order to fully comprehend each specific case. In this review, we sum up five action models concerning lignin with defense responses. Hopefully, this will help to deepen research in this field.

In the future, we suggest that studies on this subject should focus more on authentic mechanisms in each special case. Particularly, it should pay attention to different pathogens (bacteria, fungi) and ecotypes (necrotrophic, saprophytic) which will distinguish them among unique actions. The cross-talk with other defense signals needs to be considered in studies such as JA, SA, and ethylene. The metabolic flux across the different pathways should also be addressed to elucidate the mechanism behind the investigation. With more research data available, there will be more models to be uncovered, and then we will have a panoramic insight on these complicated events. This will help to develop high-resistant crops by a molecular breeding strategy.

## Data Availability

All data are contained within the article.
